# Expression of Concern: Molecular epidemiology and genetic diversity of *Anaplasma* and *Theileria* spp. in Pakistani sheep

**DOI:** 10.1371/journal.pone.0358102

**Published:** 2026-09-14

**Authors:** The PLOS One Editors

The *PLOS One* Editors issue this Expression of Concern because this article [1] was identified as one of a series of submissions for which we have concerns about the integrity and/or reliability of the peer review process.

Readers are advised to interpret the article [1] with consideration of this issue. In following up on this matter, PLOS did not evaluate the article’s integrity or scientific validity.
