# Supplementary material for: Molecular epidemiology and genetic diversity of Anaplasma and Theileria spp. in Pakistani sheep
Source: PLoS One. 2025 Jul 23;20(7):e0328364. doi: 10.1371/journal.pone.0328364 (PMC12286364; doi:10.1371/journal.pone.0328364)
Supplement: S1 Table — (DOC) [file pone.0328364.s001.doc]

**Supplementary Table 1.** Primer sequences, targeted genes, annealing temperature and expected amplicon size for the detection of *Anaplasma* spp., *Anaplasma ovis, Theileria ovis* and *Theileria* lestoquardi in sheep blood samples that were collected during present study.

| **Targeted pathogen** | **Target gene** | **Sequence 5′ to 3′** | **Annealing temperature (^o^C)** | **Amplicon size** | **Reference** |
| --- | --- | --- | --- | --- | --- |
| *Anaplasma* spp. | 16S rRNA | GGTACCYACAGAAGAAGTCC TAGCACTCATCGTTTACAGC | 52 | 345 bp | [13] |
| *Anaplasma ovis* | *msp4* | TGAAGGGAGCGGGGTCATGGG GAGTAATTGCAGCCAGGCACTCT | 62 | 347 bp | [14] |
| *Theileria ovis* | 18S rRNA | TCGAGACCTTCGGGTGGCGT TCCGGACATTGTAAAACAAA | 59 | 520 bp | [15] |
| *Theileria lestoquardi* | 18S rRNA | GTGCCGCAAGTGAGTCA GGACTGATGAGAAGACGATGAG | 55 | 785 bp | [16] |
